# From the Cochrane Library: Interventions for Impetigo

**DOI:** 10.2196/33433

**Published:** 2021-12-03

**Authors:** Ani Oganesyan, Torunn Sivesind, Robert Dellavalle

**Affiliations:** 1 Department of Dermatology University of Colorado Anschutz Medical Campus Aurora, CO United States; 2 Dermatology Service Rocky Mountain Regional VA Medical Center Aurora, CO United States

**Keywords:** impetigo, pustular lesions, Staphylococcus aureus, Streptococcus pyogenes, dermatology, skin infection

Impetigo is a contagious, superficial skin infection, most commonly affecting children, caused by *Staphylococcus aureus*, group A beta-hemolytic streptococcus (*Streptococcus pyogenes*), or both pathogens in combination [1]. Bacteria infect the epidermis, leading to itchy or painful, yellow-crusted, erythematous plaques. If blisters are present, the infection is referred to as bullous impetigo [2]. While untreated impetigo is often self-limited, treatment is important for symptom control, limiting the spread of infection and minimizing the risk of developing life-threatening complications. Due to the prevalence and risks associated with impetigo, evidence-based research to inform treatment guidelines is critical to decreasing its disease burden [1].

Current treatment options for impetigo, summarized in Table 1, include topical and systemic antibiotics, as well as topical disinfectants [2]. A 2012 Cochrane review, “Interventions for Impetigo” [2], assessed 68 randomized controlled trials (26 oral treatments and 24 topical treatments for the management of primary impetigo). Specifically, various management strategies were evaluated: watchful waiting, topical disinfectants (saline, hexachlorophene, povidone-iodine, chlorhexidine), topical antibiotics (neomycin, bacitracin, polymyxin B, gentamycin, fusidic acid, mupirocin, retapamulin, topical steroid/antibiotic combination), and systemic antibiotics (penicillin, [flu]cloxacillin, amoxicillin/clavulanic acid, erythromycin, cephalexin). Primary outcome measures included an assessment of clearance of crusts, blisters, and redness, as well as resolution of associated symptoms.

**Table 1:** Current guidelines for the management of impetigo.

Treatment		Dosing and usage	Evidence grade^a^
**Topical antibiotics**			
	Mupirocin 2% ointment	3 times daily for 5-7 days	Strong recommendation
	Retapamulin 1% ointment	2 times daily for 5 days	Strong recommendation
	Fusidic acid 2% cream	3 times daily until healed or up to 14 days	Not available in the United States
**Oral antibiotics**			
	Dicloxacillin, 250 mg; cephalexin, 250 mg	4 times daily for 7 days for empiric therapy in adults	Strong recommendation
	Cephalexin, 250 mg	4 times daily for 7 days for empiric therapy in adults	Strong recommendation
	Cephalexin, 25-50 mg/kg/day	3-4 divided doses for empiric therapy in children	Strong recommendation
	Penicillin	If culture yields streptococci alone	Strong recommendation
	Doxycycline, clindamycin, or trimethoprim-sulfamethoxazole	If methicillin-resistant *Staphylococcus aureus* (MRSA) is suspected or confirmed	Strong recommendation

^a^Recommendation according to the Infectious Diseases Society of America, using the GRADE (Grading of Recommendations, Assessment, Development, and Evaluation) system’s strength of recommendation: *strong recommendation* (desirable effects clearly outweigh undesirable effects or vice versa) and *weak recommendation* (desirable effects closely balanced with undesirable effects, or [with low- or very low–quality evidence] uncertainty in the estimates of desirable effects, harms, and burden so they may be closely balanced).

Topical antibiotics (mupirocin, retapamulin, fusidic acid) were found to be more effective than the placebo and preferable to oral antibiotics for limited impetigo. Topical antibiotics were also superior to disinfection methods. No significant differences were found in studies evaluating oral antibiotics, with the exception that penicillin was less effective than most other antibiotics. Due to insufficient evidence, the efficacy of these treatments for patients with more extensive disease could not be established. However, newer data suggest systemic antibiotics are more efficacious for patients with 5 or more lesions, or with oral or deep tissue involvement [3]. Significant findings pertaining to the treatment comparisons in this review are summarized in Table 2.

Of note, the authors of the Cochrane review pointed to a lack of evidence regarding impetigo treatment in developing countries and endemic populations—a significant data gap given that impetigo disproportionately affects children in resource-poor communities and has the highest prevalence among Australian Aboriginal children (up to 49%). A recent systematic review [4] provided much-needed insight, calling for research into topical antimicrobials for impetigo as alternatives to current first-line therapy (oral co‐trimoxazole and intramuscular benzathine penicillin G) in rural Australia. Currently, there are no trials of topical antibiotics for impetigo in high-burden settings, highlighting the need for further studies.

**Table 2:** Treatment comparison with respective results, risk ratio (RR), 95% CI, and number of studies and participants.

Comparison	Measurement	Result	Statistics
Topical antibiotic vs placebo	Investigator assessment	Topical antibiotic was superior	RR 2.24, 95% CI 1.61-3.13; 6 studies, n=575
Topical mupirocin vs topical fusidic acid	Investigator assessment	No difference	RR 1.03, 95% CI 0.95-1.11; 4 studies, n=440
Topical mupirocin vs oral erythromycin	Investigator assessment	Topical mupirocin was superior	RR 1.07, 95% CI 1.01-1.13; 10 studies, n=581
Penicillin vs erythromycin	Investigator assessment	Erythromycin was superior	RR 1.29, 95% CI 1.07-1.56; 2 studies, n=79
Penicillin vs cloxacillin	Investigator assessment	Cloxacillin was superior	RR 1.59, 95% CI 1.21-2.08; 2 studies, n=166
Topical antibiotics vs disinfecting treatments	Investigator assessment	Topical antibiotic was superior	RR 1.15, 95% CI 1.01-1.32; 2 studies, n=292

In industrialized settings, data continue to support the use of topical mupirocin and retapamulin as first-line treatments for primary impetigo. Current guidelines (Table 1) recommend topical antibiotics as the initial therapy for most patients. In patients with numerous lesions, ulceration into the dermis, or in outbreaks affecting several people, oral antibiotics are preferred [5].

The commonality of impetigo and its rapidly changing antibiotic resistance patterns make it a moving target. Its contagious nature and associated morbidity further emphasize the need for updated guidelines.
